# Digenic Inheritance of *PROKR2* and *WDR11* Mutations in Pituitary Stalk Interruption Syndrome

**DOI:** 10.1210/jc.2017-00332

**Published:** 2017-04-27

**Authors:** Shana E. McCormack, Dong Li, Yeon Joo Kim, Ji Young Lee, Soo-Hyun Kim, Robert Rapaport, Michael A. Levine

**Affiliations:** 1 Division of Endocrinology and Diabetes, Children’s Hospital of Philadelphia, Philadelphia, Pennsylvania 19104; 2 Center for Applied Genomics, Department of Pediatrics, Children’s Hospital of Philadelphia, Philadelphia, Pennsylvania 19104; 3 Molecular and Clinical Sciences Research Institute, St. George’s, University of London, Cranmer Terrace, London SW17 0RE, United Kingdom; 4 Division of Pediatric Endocrinology and Diabetes, Kravis Children’s Hospital at Mount Sinai, New York, New York 10029

## Abstract

**Context:**

Pituitary stalk interruption syndrome (PSIS, ORPHA95496) is a congenital defect of the pituitary gland characterized by the triad of a very thin/interrupted pituitary stalk, an ectopic (or absent) posterior pituitary gland, and hypoplasia or aplasia of the anterior pituitary gland. Complex genetic patterns of inheritance of this disorder are increasingly recognized.

**Objective:**

The objective of this study was to identify a genetic cause of PSIS in an affected child.

**Methods:**

Whole exome sequencing (WES) was performed by using standard techniques, with prioritized genetic variants confirmed via Sanger sequencing. To investigate the effects of one candidate variant on mutant WDR11 function, Western blotting and coimmunofluorescence were used to assess binding capacity, and leptomycin B exposure along with immunofluorescence was used to assess nuclear localization.

**Results:**

We describe a child who presented in infancy with combined pituitary hormone deficiencies and whose brain imaging demonstrated a small anterior pituitary, ectopic posterior pituitary, and a thin, interrupted stalk. WES demonstrated heterozygous missense mutations in two genes required for pituitary development, a known loss-of-function mutation in *PROKR2* (c.253C>T;p.R85C) inherited from an unaffected mother, and a *WDR11* (c.1306A>G;p.I436V) mutation inherited from an unaffected father. Mutant WDR11 loses its capacity to bind to its functional partner, EMX1, and to localize to the nucleus.

**Conclusions:**

WES in a child with PSIS and his unaffected family implicates a digenic mechanism of inheritance. In cases of hypopituitarism in which there is incomplete segregation of a monogenic genotype with the phenotype, the possibility that a second genetic locus is involved should be considered.

## Teaser

A genetic cause was sought in a child with combined multiple pituitary hormone deficiencies. The findings implicate a digenic mechanism of inheritance, with a mutation in *PROKR2* and in *WDR11*.

Pituitary stalk interruption syndrome (PSIS, ORPHA95496) is a congenital defect of the pituitary gland that is characterized by the triad of a very thin or interrupted pituitary stalk, an ectopic or absent posterior pituitary gland, and hypoplasia or aplasia of the anterior pituitary gland. Patients with PSIS may present with heterogeneous clinical features resulting from isolated or combined hypothalamic-pituitary hormone deficiencies. The rare occurrence of families in which several members have PSIS has suggested that at least in some cases PSIS may be genetic (1, 2), and mutations in genes encoding proteins involved in the Wnt, Notch, and Shh signaling pathways that are critical for hypothalamic–pituitary development have been reported in some patients with PSIS (3). Nevertheless, in most cases the cause of PSIS remains unknown. We present a case of PSIS with multiple anterior pituitary deficiencies in which whole exome sequencing (WES) analysis identified two heterozygous mutations, thereby implicating a digenic mechanism as the basis for this condition.

## Materials and Methods

### Human subject considerations

Samples were collected under an approved institutional review board protocol of the Children’s Hospital of Philadelphia; the corresponding study was conducted according to the Declaration of Helsinki. Written, informed consent or assent, as appropriate, was obtained from all participants before their inclusion.

### Case presentation

Relevant clinical details were abstracted from the electronic medical record.

### Biochemical and genetic analyses

**Figure 1.**
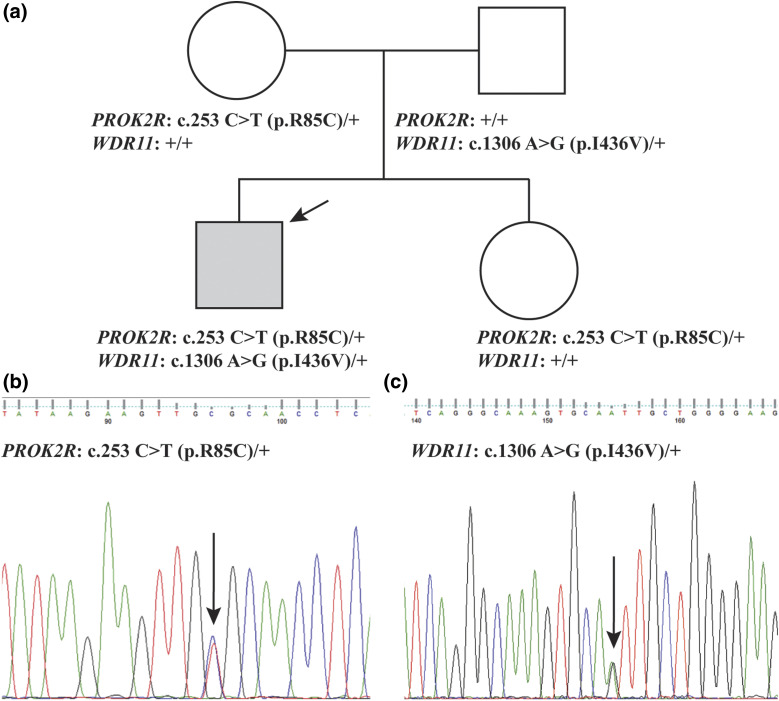
Pedigree and sequencing chromatograms. (a) Pedigree: The affected proband is indicated with an arrow (gray square). The genotypes are indicated for each individual. (b) A sequence chromatogram showing the *PROKR2* (c.253C>T;p.R85C) mutation. (c) A sequence chromatogram showing the *WDR11* (c.1306A>G;p.I436V) mutation. In the chromatograms, mutations are indicated with arrows.

We extracted DNA from peripheral blood mononuclear cells from the proband and his unaffected parents, performed exome capture and sequencing, and read processing, mapping to human genome reference (GRCh37-derived alignment set used in 1000 Genomes Project), variant calling, annotations, and filtering for rare variants affecting the coding sequence and/or consensus splice sites, as previously described (4). The family pedigree (Fig. 1) did not suggest a specific mode of inheritance, so we considered nonsynonymous, splice-altering variants, and frameshift variants cosegregating with the disease under *de novo* dominant or recessive inheritance model in the family with a minor allele frequency <1% in public databases (*i.e.,* 1000 Genomes Project and National Heart, Lung, and Blood Institute ESP6500SI). Subsequent gene prioritization was on the basis of deleterious prediction, biological, and clinical relevance, as suggested by existing databases (*i.e.*, Online Mendelian Inheritance in Man and Human Gene Mutation Database). However, because we failed to identify any candidates relevant to the phenotype under either the dominant or the recessive model, we next focused on a possible digenic mode of inheritance by identifying variants in the proband that were shared with either parent. We validated mutation candidates by Sanger sequencing and used *in silico* tools (5, 6) to predict their effects. Deletion/duplication testing was also performed in *PROKR2* by using standard techniques (Fulgent Diagnostics, Temple, CA). Salivary DNA was collected from the unaffected sister to assess her status for the identified variants. All biochemical analyses were performed in commercial reference laboratories using standard techniques.

### Mutagenesis

The full-length *WDR11* complementary DNA in pcDNA–green fluorescent protein (GFP) or pcDEST-Myc vector was mutagenized by using a Q5 site-directed mutagenesis kit (New England BioLabs, Ipswich, MA) to introduce the c.1306A>G variant, following the manufacturer’s protocol. Briefly, the nucleotide exchange was introduced by using Q5 Hot Start High-Fidelity DNA Polymerase with “nonoverlapping” mutagenic primers (NEBase Changer; New England BioLabs) via polymerase chain reaction run for 25 cycles of 98°C for 30 seconds, 98°C for 10 seconds, 60°C for 30 seconds, 72°C for 5 minutes, and 72°C for 2 minutes. The products were ligated at room temperature for 5 minutes and transformed into Top10 chemically competent cells [One Shot Top10 chemically competent *Escherichia coli* (Thermo Fisher Scientific, Hertfordshire, United Kingdom)]. Mutated plasmid constructs were verified by sequencing (Source Bioscience, Nottingham, United Kingdom). Sequences of primers used are available upon request.

### Coimmunoprecipitation and Western blot analysis

HEK293T cells cultured in Dulbecco’s modified Eagle medium supplemented with 2 mM l-glutamine, 100 µg/mL penicillin/streptomycin, and 10% fetal bovine serum (Sigma-Aldrich, St. Louis, MO) were transfected with plasmid constructs expressing Myc-tagged WDR11 or hemagglutinin-tagged EMX1 using Fugene (Promega, Madison, WI). At 48 hours after transfection, total cell lysates were extracted with the lysis buffer (50 mM HEPES, 150 mM NaCl, 10% glycerol, 1% Nonidet P-40, and 1 mM EDTA) containing protease inhibitor cocktail (Sigma-Aldrich) and phosphatase inhibitor (Sigma-Aldrich). After incubation on ice for 10 minutes and centrifugation for 10 minutes at 4°C, the precleared lysate (500 µg – 1 mg protein) was incubated with anti-Myc antibody (M4439, Sigma-Aldrich) and protein A/G-Agarose beads (Santa Cruz Biotechnology, Dallas, TX) overnight at 4°C on a rotating wheel. The immune complexes on the beads were washed four times with the lysis buffer, separated by sodium dodecyl sulfate–polyacrylamide gel electrophoresis and transferred to Hybond-ECL membrane (Amersham, GE Life Sciences, Marlborough, MA), which was probed with anti-WDR11 (1:500, ab175256, Abcam) and anti-EMX1 (1:500, PA5-35373; Invitrogen, Carlsbad, CA) antibody diluted in blocking buffer (5% skim milk in Tris-buffered saline with 0.05% Tween 20).

### Immunofluorescence

GFP-tagged wild-type (WT) and mutant *WDR11* expression constructs were transfected into HEK293T. After 48 hours, cells were treated with leptomycin B (10 ng/mL) for 10 hours before being fixed with 4% paraformaldehyde. After being washed three times in phosphate-buffered saline, the nuclei were counterstained with 4′,6-diamidino-2-phenylindole (DAPI) and the cover slips were mounted in Mowiol 4-88 (Fluka; Sigma-Aldrich). Images were analyzed by using Zeiss Axioplan 2 Upright fluorescence microscope (Carl Zeiss, Oberkochen, Germany) and ImageJ software (National Institutes of Health, Bethesda, MD). In each experiment, approximately 200 cells were scored for the nuclear or cytoplasmic location of WDR11 based on the GFP signal at 488 nm, against the total cells in the field based on the DAPI signal at 405 nm.

## Results

The proband was the 2.92-kg product of a 39-week gestation, born to a 45-year-old gravida 2, para 1, abortion 0, mother (Fig. 1). He is of Ashkenazi Jewish heritage and was conceived by *in vitro* fertilization and underwent targeted preimplantation genetic diagnosis because both parents are carriers for Gaucher disease and familial hyperinsulinism. He is a carrier of *GBA* (MIM 606463; GenBank: NM_001005741.2; rs7673715) c.1226A>G; p.N409S and *ABCC8* (MIM 600509; NM_000352.4; rs151344623) c.3989-9G>A mutations.

He developed transient, mild hypoglycemia within the first 36 hours of life and was diagnosed with central congenital hypothyroidism on the basis of low thyroid hormone levels on newborn screening. Magnetic resonance imaging of the brain showed small anterior pituitary and ectopic posterior pituitary with a thin and interrupted pituitary stalk, consistent with PSIS. Hydrocortisone replacement was begun at 6 weeks of life based on low serum cortisol levels of ∼1 to 2 μg/dL. At birth he was thought to have a normal-sized phallus, but at 8 months of life he received testosterone, 25 mg intramuscularly every 3 weeks × 6, for small phallus.

Growth hormone status was initially normal, and his length was in the 25th to 50th percentile until age ∼16 to 18 months, after which he made little gain in length, such that by age 2 8/12 years, his height was below the first percentile. His head circumference demonstrated a similar pattern. His weight increased to within the 50th to 75th percentile by around age 12 months but also faltered thereafter. He begun receiving recombinant human growth hormone (GH) at age 2 9/12 years because of lack of statural growth (height standard deviation, −1.8), a very low random growth hormone level of 0.93 ng/mL, low insulin-like growth factor-1 level of 27 ng/mL, and a low insulin-like growth factor-binding protein 3 level of 0.6 mg/L, all of which in this clinical context were consistent with GH deficiency. Both height and weight responded well to GH; height increased to the 38th percentile and weight to the 20th percentile after ∼15 months of therapy. Neurocognitive development has been normal.

WES revealed heterozygous mutations in two genes known to affect hypothalamic and pituitary development: c.253C>T;p.R85C in *PROKR2* (MIM 607123; NM_144773.2; rs141090506) inherited from an unaffected mother and c.1306A>G;p.I436V in *WDR11* (MIM 606417; NM_018117.11; rs34602786) inherited from an unaffected father, both confirmed by Sanger sequencing (Fig. 1). Additional candidates were considered under *de novo*, X-linked, and recessive models and were excluded because of a lack of pathogenicity or relevance to the phenotype (Supplemental Table 1). A clinically unaffected sister carried only the *PROKR2* missense mutation (Fig. 1). Publicly available databases show that *PROKR2* missense mutation is present in the population with a minor allele frequency (MAF) of 0.0005024 in the ExAC database (http://exac.broadinstitute.org/gene/ENSG00000101292) and is absent in the 1000 Genomes Project and in 6503 exomes from the Exome Sequencing Project (ESP6500SI).

We were unable to identify a point mutation, deletion, or duplication in the second *PROKR2* allele, and therefore the *WDR11* p.I436V variant became relevant on the basis of the involvement of *WDR11* mutations in other pituitary disorders. This variant is rare, and publicly available databases show it has a MAF of 0.001307 in the ESP6500SI (http://evs.gs.washington.edu/EVS/) and a MAF of 0.00090840 in the ExAC database (http://exac.broadinstitute.org/gene/ENSG00000120008). Although *in silico* prediction tools do not indicate a high likelihood of pathogenicity (PolyPhen-2 score of 0.000; PROVEAN score of −0.118, neutral), its location in the functionally significant sixth WD domain, an important site for protein–protein interactions, suggested it could be pathogenic.

**Figure 2.**
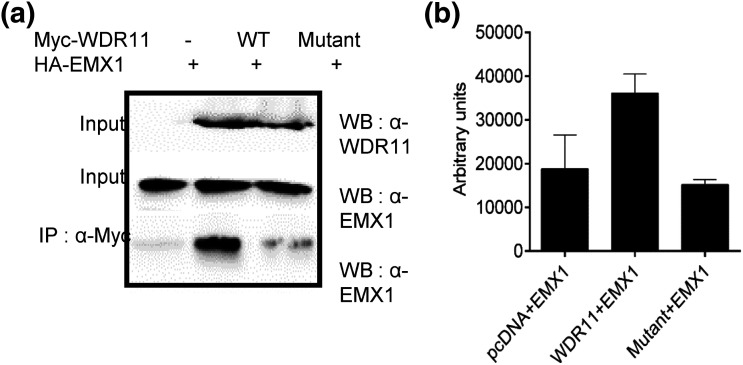
Mutant WDR11 fails to bind to EMX1. (a) WDR11 fusion proteins containing a Myc epitope tag WT or the c.1306A>G variant (mutant) were coexpressed in HEK293 cells along with HA-EMX1 protein. The total cell lysates were immunoprecipitated with anti-Myc antibody, and the association of EMX1 protein was determined by immunoblot analysis using anti-EMX1 antibody. Empty pcDNA vector (-) was included as a negative control. (b) The average densitometry values of the EMX1 band intensity obtained from three independent experiments are shown with the standard deviations (error bars).

**Figure 3.**
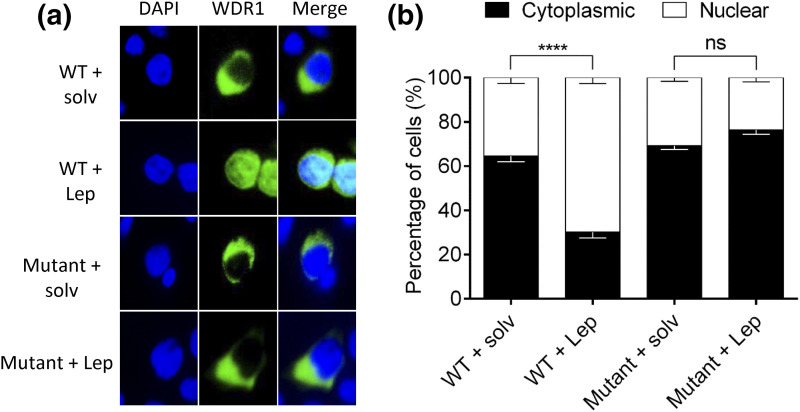
Mutant WDR11 fails to accumulate in the nucleus after treatment with leptomycin B. (a) HEK293 cells transfected with GFP-WDR11 (green) expression constructs were treated with leptomycin B (Lep), an inhibitor of nuclear export, or the vehicle (solv) and analyzed by fluorescence microscopy to determine the intracellular localization of WDR11. Nuclei were stained with DAPI (blue). (b) The percentages of cells showing nuclear or cytoplasmic localization of the mutant WDR11-GFP are shown in comparison with the WT, which showed a significant increase of nuclear translocation after Lep treatment. The average data from three independent experiments were obtained by counting 100 to 200 cells in each experiment and are presented as mean ± standard error of the mean with two-way analysis of variance followed by Tukey post hoc test. NS, *P* > 0.05; ****P < 0.0001.

To confirm this, we examined the behavior of this rare variant of *WDR11* in two different functional assays *in vitro*. WDR11 binds and colocalizes with the EMX1 transcription factor in the nucleus, and this interaction must be important for its normal function because this capacity is lost by some, although not all, *WDR11* mutations in patients with idiopathic hypogonadotropic hypogonadism and Kallmann syndrome (7). Our coimmunoprecipitation Western blot assay indicated that the p.I436V variant of WDR11 was unable to bind to EMX1 (Fig. 2). We also evaluated the p.I436V variant in a second functional assay that is based on the observation that WT WDR11 can shuttle between nucleus and cytoplasm, and treatment with a nuclear export inhibitor, leptomycin B, induces its accumulation in the nucleus (7). When we introduced GFP-WDR11 into HEK293T cells, both the WT and variant WDR11 proteins showed mainly cytoplasmic location, but when the cells were treated with leptomycin B, the WT but not the p.I436V variant showed nuclear localization (Fig. 3). Taken together, these studies demonstrate that the p.I436V substitution disrupts the normal function of WDR11 and provides strong evidence that this is a pathogenic variant.

## Discussion

PSIS is a common finding in patients with pituitary hormone deficiency and accounts for hypopituitarism in >11% of adult patients (8) and in 29 of 46 children with idiopathic growth hormone deficiency (9). Although mechanical pituitary stalk rupture or pituitary stalk ischemia during breech delivery has been implicated as a major cause of PSIS, the existence of familial cases has suggested that a genetic disorder involving developmental processes underlies at least some cases of PSIS, and mutations and/or single nucleotide variants in *HESX1* (MIM 601802), *LHX4* (MIM 602146), *PROP1* (MIM 601538), *OTX2* (MIM 600037), *SOX3* (MIM 313430), *PROKR2* (MIM 607123), and *GPR161* (MIM 612250) have been identified in patients with this condition (2, 3, 10). Our understanding of the genetic basis of this disease continues to grow; complex inheritance patterns and interpreting the significance of variation are two important challenges (2). Despite the increasing availability of high-throughput genetic sequencing techniques, most patients with PSIS do not have an identified genetic cause. Here, we have used an unbiased approach, WES, to identify a genetic basis for PSIS. The proband we studied carried heterozygous missense mutations in two different genes important for hypothalamic/pituitary function, *PROKR2* and *WDR11*. This finding implicates a putative digenic basis for PSIS in this child and also suggests that this unusual genetic mechanism may explain other cases of PSIS that lack conventional autosomal-recessive inheritance.

A digenic disorder results from heterozygous mutations in two distinct genes that encode different proteins. Often, these proteins are both required for normal function or development of a tissue, and/or act in the same signaling pathway. The effect of having mutations in two different genes in the same pathway can be more than additive; together they can produce a more severe phenotype than would be expected to occur from the simple combination of their individual effects. For a long time, the existence of digenic inheritance has been proposed as one potential explanation for why in some pedigrees, a monogenic model of inheritance suggests decreased or variable penetrance (11). In these families, a two-locus model may more accurately reflect the observed patterns. Examples of digenic inheritance include some forms of retinitis pigmentosa and facioscapulohumeral muscular dystrophy and, perhaps more relevantly, in some forms of idiopathic hypogonadotropic hypogonadism (12). The number of conditions exhibiting digenic inheritance continues to grow (13). Indeed, a curated database exists [DIDA (DIgenic Diseases Database)] and at the time of this writing contains 44 conditions with clear evidence of digenic inheritance (14).

Although we report on heterozygous mutations in both *PROKR2 and WDR11* in the same individual, each of these genes has previously been implicated with another gene mutation as the basis for a digenic pituitary disease. *PROKR2* encodes a 384-amino acid G-protein–coupled receptor (GPCR) whose signaling activity plays a key role in both development of the olfactory bulb (15) and gonadotropin-releasing hormone secretion (16). Mutations in *PROKR2* have previously been associated with hypogonadotropic hypogonadism with or without anosmia and Kallmann syndrome (17).

Several lines of evidence indicate that *PROKR2* p.R85C is pathogenic. First, the amino acid change (from basic to hydrophobic) occurs at a highly evolutionarily conserved site that is predicted to lie within an important functional G-protein–coupled receptor domain, and *in silico* studies predict that this change is pathological (PolyPhen-2 score of 1.0 predicts the change is probably damaging; PROVEAN score of −6.840 is deleterious). Second, functional studies have indicated that the p.R85C variant has reduced activity in mitogen-activated protein kinase and/or calcium signaling pathways (17–19) without evidence of a dominant negative effect (19). Third, this mutation has previously been identified in an individual with normosomic idiopathic hypogonadotropic hypogonadism (17). The same heterozygous mutation on *PROKR2* that was identified in the proband here, c.253C>T;p.R85C, has also been found in a female patient with combined pituitary hormone deficiencies, including in GH, adrenocorticotropic hormone, luteinizing hormone, and follicle-stimulating hormone, as well as vasopressin, and magnetic resonance imaging demonstrating normal anterior pituitary, absent posterior pituitary, and absent stalk (20). No additional mutations were found in that patient; however, only two candidate genes [*PROKR2* and *FGFR1* (MIM 136350)] were sequenced for individuals in that study. Finally, all other amino acid changes reported at the same site have been also associated with hypothalamic and pituitary dysfunction. For example, heterozygous mutations, including *PROKR2* c.254G>A;p.R85H (rs74315418), c.253C>G;p.R85G (rs141090506), and c.254G>T;p.R85L (rs74315418), have all been identified in association with pituitary stalk interruption and combined pituitary hormone deficiencies (21, 22). On the basis of the American College of Medical Genetics and Genomics guidelines (23), this variant is therefore classified as pathogenic.

In addition, in the mouse model of PROKR2 deficiency, the Kallmann syndrome phenotype is observed only in the homozygous animals (18). In human studies as well as in animal models, *PROKR2* haploinsufficiency seems not to account for the PSIS phenotype, hence raising the need for a “second hit,” either genetic or environmental, to produce pituitary disease. This hypothesis was further supported when a patient with Kallmann syndrome was discovered to carry the same *PROKR2* heterozygous mutation as our proband, p.R85C, in combination with a second heterozygous mutation in *FGFR1*, c.1810G>A;p.A604T (NM_023110.2), thereby providing evidence for a digenic basis for the syndrome (22). Prokineticin 2 and PROKR2 are both expressed in the hypothalamus and pituitary, and reduced expression or activity of PROKR2 is implicated in both Kallmann syndrome and PSIS, perhaps because of the important role this signaling pathway plays in endocrine angiogenesis and neuronal migration in this region of the central nervous system. Ectopic posterior pituitary has been proposed to be a consequence of defective neuronal axon projections along the pituitary stalk or defective angiogenesis of hypophyseal portal circulation. Therefore, it is reasonable to suggest that the loss of PROKR2 signaling is involved in PSIS.

Our proband carried a second heterozygous mutation in *WDR11*, which encodes a protein that is a member of the WD repeat protein family that participates in a wide variety of cellular processes. The p.I436V missense mutation affects the sixth WD domain of the WRD11 protein within the evolutionarily conserved predicted propeller region that is required for interaction of WDR11 with EMX1, a homeodomain transcription factor necessary for olfactory bulb morphogenesis. We found that the p.I436V mutation, similar to other nearby amino acid substitutions that have been identified in individuals with idiopathic hypogonadotropic hypogonadism with and without anosmia (7, 24), disrupted interaction of WDR11 with EMX1. Specifically, heterozygous missense mutations in neighboring residues A435 (c.1303G>A;p.A435T; rs318240760) and R448 (c.1343G>A;p.R448Q; rs144440500) have been identified in each of two individuals with idiopathic hypogonadotropic hypogonadism and normosmia (7). In addition, in a different individual, the *WDR11* p.A435T mutation was identified in association with a mutation in a second gene, *GNRHR* (c.275T>C;p.L92P; MIM 138850; NM_000406.2), implicating digenic inheritance of this disorder as well (24). This variant has been identified in homozygous form in two people (http://exac.broadinstitute.org/gene/ENSG00000120008), but their phenotype is unknown, and it is conceivable that one or both of these individuals has a mild pituitary phenotype.

Digenic inheritance is more likely when the functional roles of the two involved proteins affect a singular pathway and/or there are demonstrated protein-protein interactions (13). Here, both *PROKR2* and *WDR11* participated in key signaling processes that influence the morphogenesis of the olfactory bulb, although they are not in the same singular pathway, nor do they have an established protein–protein interaction. The *PROKR2* p.R85C variant has been described in a heterozygous state in patients with idiopathic hypogonadotropic hypogonadism, Kallmann syndrome, healthy first-degree relatives of Kallmann probands, and rare healthy controls. We therefore propose that p.R85C (and possibly other *PROKR2* mutations) may act as a modifier and contribute to the PSIS phenotype through digenic inheritance, as previously demonstrated in idiopathic hypogonadotropic hypogonadism and Kallmann syndrome (12, 25). Further studies are needed to elucidate in more detail the role of PROKR2 signaling in pituitary and midline development.

In summary, this case adds digenic inheritance of mutations in *PROKR2* and *WDR11* as a potential cause of combined pituitary hormonal deficiencies and PSIS, and it highlights the importance of considering unconventional genetic mechanisms when there is incomplete segregation of a heterozygous mutation with the phenotype in a pedigree.
